# Onset of relief of dyspnoea with budesonide/formoterol or salbutamol following methacholine-induced severe bronchoconstriction in adults with asthma: a double-blind, placebo-controlled study

**DOI:** 10.1186/1465-9921-7-141

**Published:** 2006-12-04

**Authors:** René E Jonkers, Theo A Bantje, René Aalbers

**Affiliations:** 1 Department of Pulmonary Diseases, Academic Medical Centre, Amsterdam, The Netherlands; 2 Department of Pulmonary Diseases, Amphia Ziekenhuis, Breda, The Netherlands; 3 Department of Pulmonary Diseases, Martini Hospital, Groningen, The Netherlands

## Abstract

**Background:**

The long-acting β_2_-agonist (LABA) formoterol has an onset of effect comparable to that of salbutamol. Consequently, the combination of formoterol and budesonide in one inhaler, approved for maintenance use, can potentially be used for reliever therapy. This study compared the onset of relief from induced bronchospasm with a single dose of budesonide/formoterol versus standard salbutamol therapy in patients with asthma.

**Methods:**

In this randomised, double-blind, placebo-controlled, cross-over study, 32 patients with asthma underwent a methacholine provocation test leading to a fall in forced expiratory volume in 1 second (FEV_1_) of ≥30% at enrolment (Visit 1) and three subsequent study visits (Visits 2–4). Immediately after each provocation at Visits 2–4, patients received one of three test treatments: one inhalation of budesonide/formoterol 160/4.5 μg (via Turbuhaler^®^), two inhalations of salbutamol 100 μg (via a pressurised metered-dose inhaler [pMDI]) or placebo. All patients received each of the test treatments in a randomised order, after separate methacholine provocations. The effect of treatment on FEV_1 _and breathlessness (using the Borg scale) was measured at 1, 3, 5, 10, 15, 20, 25 and 30 minutes after test treatment.

**Results:**

Following methacholine provocation, Borg score increased from a baseline value of below 0.5 to 3.03, 3.31 and 3.50 before treatment with budesonide/formoterol, salbutamol and placebo, respectively. Budesonide/formoterol and salbutamol reversed methacholine-induced dyspnoea (breathlessness) rapidly. At 1 minute after inhalation, statistically significant decreases in Borg score were observed for budesonide/formoterol and salbutamol (p = 0.0233 and p < 0.0001, respectively, versus placebo), with similar rapid increases in FEV_1 _(both active treatments p < 0.0001 versus placebo). The median time to 50% recovery in Borg score after methacholine provocation was 3 minutes with budesonide/formoterol, 2 minutes with salbutamol and 10 minutes with placebo. All treatments and procedures were well tolerated.

**Conclusion:**

Single doses of budesonide/formoterol and salbutamol both provided rapid relief of dyspnoea and reversal of severe airway obstruction in patients with asthma with experimentally induced bronchoconstriction. The perception of relief, as confirmed by objective lung function assessment, provides evidence that budesonide/formoterol can be used as reliever medication in asthma.

## Background

For many years, short-acting β_2_-agonists (SABA), such as salbutamol and terbutaline, have played an important role in the treatment of asthma; the bronchodilating effects have, indeed, proved to be life-saving for episodes of acute asthma. The long-acting β_2_-agonist (LABA) formoterol has a comparable onset of action to salbutamol and terbutaline, based upon objective lung measurements such as forced expiratory volume in 1 second (FEV_1_) [1-7]. Furthermore, as-needed formoterol provides superior asthma control compared with terbutaline [6] and salbutamol [7,8]. The rapid onset of action and long duration of effect of formoterol are now acknowledged in asthma treatment guidelines [9].

The combination of budesonide and formoterol in one inhaler improves asthma control compared with a similar or higher dose of inhaled corticosteroid (ICS) [10-13]. Moreover, the rapid onset of effect of formoterol suggests that budesonide/formoterol is suitable for both maintenance and reliever therapy, i.e. without the need for a separate SABA. Clinical studies show that use of budesonide/formoterol for both maintenance and reliever therapy provides additional improvements in asthma control (assessed by symptoms and exacerbations) over the same maintenance therapy plus SABA for relief [14,15]. The effectiveness of this novel regimen, where patients use budesonide/formoterol as their only reliever medication, is thought to be the result of early intervention with rapid increases in ICS dose at the first signs of symptoms [16,17].

One potential concern with as-needed budesonide/formoterol use is that patients switching from a SABA to budesonide/formoterol as reliever medication may fail to achieve similarly rapid relief of their symptoms. The efficacies of high-dose formoterol [5,18] and budesonide/formoterol [19,20] have been demonstrated in patients with acute asthma. To date, however, no studies have demonstrated whether the lowest dose of formoterol in budesonide/formoterol (i.e. 160/4.5 μg administered via the dry-powder inhaler Turbuhaler^®^), provides a similar onset of efficacy as a standard dose of salbutamol in a situation of acute severe bronchospasm. This was assessed in the present study, which compared the onset of effect of a single dose of budesonide/formoterol with two 100 μg inhalations of salbutamol (administered via a pressurised metered-dose inhaler [pMDI]) for relieving dyspnoea (breathlessness) in patients with acute asthma symptoms provoked by methacholine challenge.

## Methods

### Study population

Patients were required to fulfil the following inclusion criteria: male or female outpatients aged between 18 and 50 years (inclusive), with asthma for a minimum of 6 months (American Thoracic Society definition [21]) prior to Visit 1; a baseline FEV_1 _of > 1.5 L and > 60% of predicted normal [22]; a provocative concentration of methacholine causing a 20% fall in FEV_1 _(PC_20_-MCh) ≤ 8 mg/mL and a demonstrated fall in FEV_1 _of > 30% upon continuation of the provocation test; ability to inhale correctly through Turbuhaler^® ^and pMDI inhalers.

Patients were excluded from the study if, within 6 weeks prior to Visit 1, they had used oral, rectal or intravenous corticosteroids or if they had experienced an asthma exacerbation or a change in ICS dose. Female patients who were pregnant, planning pregnancy, breastfeeding or not using an adequate method of contraception (as judged by the investigator) were also excluded. Other exclusion criteria included the use of β-blocker therapy (including eye drops) and any significant disease or disorder that might either put the patient at risk because of participation in the study or negatively influence the patient's ability to participate in the study.

Patients were asked to avoid strenuous exercise for 2 hours, smoking for 1 hour and consumption of caffeine-containing beverages for 8 hours before clinic visits (Visits 1–4) and until all study-related procedures had been completed at the visit.

The study protocol and informed consent form were approved by an independent ethics committee. The study was performed in accordance with the Declaration of Helsinki. Informed consent was obtained from all patients.

### Study design

This randomised, double-blind, double-dummy, placebo-controlled, crossover study (study code D5890C0007) was conducted at three centres in The Netherlands. The study comprised an initial enrolment visit (Visit 1) and three study visits (Visits 2, 3 and 4), with each visit separated by 3–14 days. Prior to each visit patients were required to withdraw from bronchodilator medication. At each visit patients underwent a methacholine provocation test, using the 2-minute tidal breathing method [22], leading to a fall from baseline in FEV_1 _of ≥ 30%. The methacholine test was only performed if the patient's FEV_1 _at baseline, prior to commencing the test, was > 1.5 L, differed by not more than ± 15% from the Visit 1 value and was > 60% of predicted normal. The same methacholine provocation method was used in all centres.

Patients were randomised at Visit 2. At Visits 2–4, immediately following the methacholine provocation test, active and double-dummy placebo treatments were administered in accordance with the randomisation schedule: one inhalation of budesonide/formoterol 160/4.5 μg (via Turbuhaler^®^), two inhalations of salbutamol 100 μg (via pMDI) or placebo. Half of the patients took their first inhalation from the Turbuhaler^®^, the other half used the pMDI first. All test medications were inhaled within 1 minute of the last methacholine dose. FEV_1 _was measured and patients graded their breathlessness using the Borg scale [23,24] during the provocation test and at 1, 3, 5, 10, 15, 20, 25 and 30 minutes after administration of the study drug.

### Methods of assessment

FEV_1 _was measured by spirometry according to the European Respiratory Society guidelines [25]. The Borg score was used to provide a measure of patients' perception of dyspnoea [24,25]. Patients were instructed on how to perform the Borg score assessment and then asked (in Dutch): 'Please indicate the level of breathlessness that you are feeling at this exact moment by choosing the appropriate number on the scale in front of you'. Patients estimated the intensity of breathlessness by selecting a score ranging from 0 to 10, with 0 indicating no appreciable breathlessness and 10 indicating maximal tolerable sensation.

Adverse events, both spontaneously reported and in response to two standard questions ('Have you had any health problems since the last visit?' and 'Have you had any health problems since you were last asked?'), were assessed at Visits 2–4, upon arrival at the clinic and before departure (after the methacholine provocation test).

### Statistical analysis

The primary efficacy outcome variable was the change in Borg score, which was defined as the difference between the Borg score obtained at the end of the methacholine provocation test (before drug intake) and the Borg score obtained at 1 minute after drug administration. Secondary outcome variables included change in FEV_1 _measurement at 1 minute, time to recovery in Borg score (50% decrease from the post-methacholine value) and time to recovery in FEV_1 _(return to 85% of the baseline FEV_1 _value).

The statistical analysis was performed at AstraZeneca R & D Lund, Sweden using Gauss from Aptech Systems Inc. and the Riemann Library (Gauss kernel revision 6.0.40; Riemann Library version 2.3.0). The mean change in Borg score from the end of the provocation test to 1 minute after study drug administration was analysed using an additive analysis of variance model, with treatment, period and patient as fixed factors and with the Borg score before drug intake (i.e. obtained at the end of the provocation test) as a covariate. Mean changes in Borg score were estimated and 95% confidence intervals were calculated.

The mean change in FEV_1 _was expressed as the ratio between the FEV_1 _1 minute after study drug administration and the FEV_1 _before drug intake (i.e. from the end of the provocation test). A multiplicative analysis of variance model with patient, period and treatment as fixed factors and FEV_1 _before drug intake as a covariate was used to analyse the mean change in FEV_1_. Geometric mean ratios in FEV_1 _were estimated and 95% confidence intervals were calculated.

Times to recovery in Borg score and FEV_1 _after drug administration were illustrated graphically using the Kaplan-Meier technique. Statistical analyses of time to recovery for Borg score and FEV_1 _were performed in separate Cox proportional hazards models stratified by patient and with treatment as factor. In addition, pairwise comparisons were performed using the Wilcoxon signed rank test. Recovery times were interpolated from the measurements of Borg score and FEV_1 _after drug administration. Adverse events were described using frequency and percentages.

## Results

**Figure 1:**
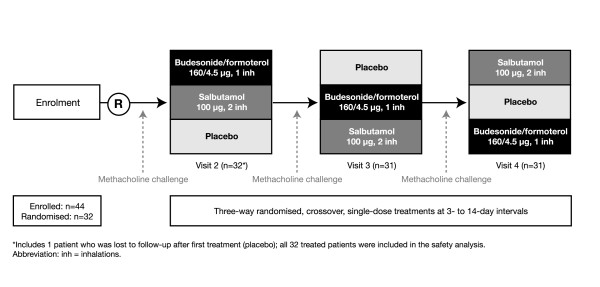
Study design and patient flow.

A total of 44 patients were enrolled in the study, 32 of whom were randomised to treatment. The safety analysis included all 32 randomised patients; however, one patient (lost to follow-up) completed only one period of treatment (placebo), hence the efficacy analysis is based on 31 patients. The study design and a summary of patient flow throughout the study are shown in Figure 1. A summary of demographic and clinical data for the 32 randomised patients is presented in Table 1.

**Table 1:** Patient baseline demographics

**Characteristic**	**Patients (n = 32)**
Sex	
Male	15
Female	17
Mean age, years [range]	33.5 [18–50]
Median time since asthma diagnosis, years [range]	14 [1–48]
ICS use	
Patients, n	29
Mean daily dose, μg [range]	677 [100–2000]
Mean FEV_1_, L [range]	3.40 [1.83–5.22]
Mean FEV_1_, % predicted normal [range]	93.6 [61–126]
Mean^a ^PC_20_, mg/mL [range]	0.47 [0.1–6.7]

^a^Geometric mean.

Abbreviations: ICS = inhaled corticosteriod, FEV_1 _= forced expiratory volume in 1 second; PC_20 _= provocative concentration of methacholine causing a 20% fall in FEV_1_.

### Efficacy

**Figure 2:**
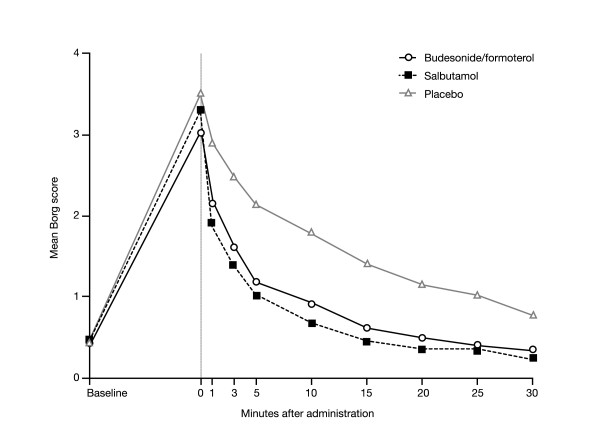
Mean Borg score for patients with asthma after methacholine challenge (time = 0) and at various timepoints after one inhalation of one of the following as reliever medication: budesonide/formoterol 160/4.5 μg (via Turbuhaler^®^), salbutamol 100 μg (via pressurised metered-dose inhaler [pMDI]) or placebo.

Borg dyspnoea score increased from a baseline value of below 0.5 to post-provocation values of 3.03, 3.31 and 3.50 before administration of budesonide/formoterol, salbutamol and placebo, respectively. A trend towards a smaller increase in Borg score was observed on the budesonide/formoterol days as compared with the salbutamol and placebo days (Figure 2; Table 2). Both budesonide/formoterol and salbutamol reversed methacholine-induced dyspnoea rapidly. At 1 minute after inhalation, a greater decrease in Borg score was observed for both budesonide/formoterol and salbutamol compared with placebo (-0.89 and -1.31 versus -0.46, respectively; p = 0.0233 and p < 0.0001, respectively, versus placebo). A statistically significant difference in favour of salbutamol was observed between the two active treatments at the 1-minute observation (mean change -0.41 for salbutamol versus budesonide/formoterol; p = 0.024).

**Table 2:** Methacholine provocation test data

Assessment	Budesonide/formoterol (n = 31)	Salbutamol (n = 31)	Placebo (n = 31)
FEV_1_, L [range]			
Before provocation	3.27 [1.78–4.93]	3.22 [1.83–4.98]	3.25 [1.83–5.11]
After provocation	2.14 [1.24–3.40]	1.99 [1.10–3.13]	2.03 [1.05–3.49]
PC_20_, mg/ml [range]	0.42 [0.08–5.81]	0.44 [0.06–4.66]	0.46 [0.07–8.64]
Borg score^a ^after provocation [range]	3.03 [1.0–5.0]	3.31 [0.5–7.0]	3.50 [0.5–7.0]

All data are presented as geometric means, apart from Borg scores, which are arithmetic means.

^a^Borg dyspnoea scores subjectively measure perceived breathlessness on a scale 0–10, where 0 = nothing at all and 10 = maximal breathlessness.

Between 3 and 30 minutes post-treatment, mean Borg scores decreased at a similar rate with budesonide/formoterol and salbutamol. A spontaneous slow recovery in Borg score was observed following inhalation of placebo, but placebo scores were always approximately double those seen after inhalation of budesonide/formoterol and salbutamol.

**Figure 3:**
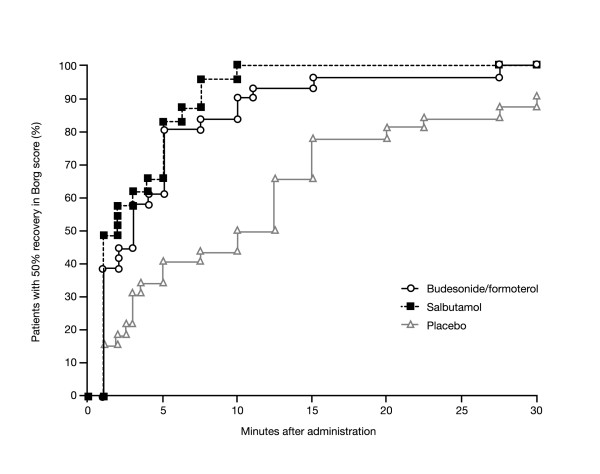
Kaplan-Meier plot for time to 50% recovery from methacholine-provoked increases in Borg dyspnoea score in patients with asthma taking one inhalation of one of the following for reliever medication: budesonide/formoterol 160/4.5 μg (via Turbuhaler^®^), salbutamol 100 μg (via pressurised metered-dose inhaler [pMDI]) or placebo. Reliever medication was given immediately after methacholine challenge.

The median time to 50% recovery in Borg score was similar for budesonide/formoterol and salbutamol (3 and 2 minutes, respectively; p = 0.1413), and significantly longer for placebo (10 minutes; p = 0.0028 and p < 0.0001 for budesonide/formoterol and salbutamol, respectively, versus placebo) (Figure 3).

**Figure 4:**
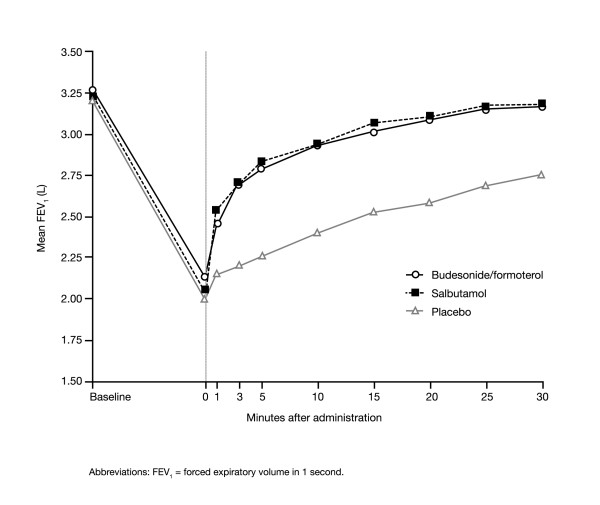
Mean FEV_1 _for patients with asthma after methacholine challenge (time = 0) and at various timepoints after one inhalation of one of the following as reliever medication: budesonide/formoterol 160/4.5 μg (via Turbuhaler^®^), salbutamol 100 μg (via pressurised metered-dose inhaler [pMDI]) or placebo.

FEV_1 _decreased rapidly from a pre-provocation value of approximately 3.25 L to approximately 2 L in all three treatment periods during methacholine provocation (Table 2; Figure 4). The reduction in FEV_1 _was smaller before administration of budesonide/formoterol compared with salbutamol or placebo. Budesonide/formoterol and salbutamol increased FEV_1 _after 1 minute versus placebo (19% and 25% versus 6%, respectively; both p < 0.0001) (Figure 4). This corresponds to a difference of 0.30 L for budesonide/formoterol and 0.39 L for salbutamol versus placebo; the corresponding difference between budesonide/formoterol and salbutamol was 0.09 L (p = 0.0309). Over the entire remaining recovery period, similar increases in FEV_1 _were seen with the two active treatments.

**Figure 5:**
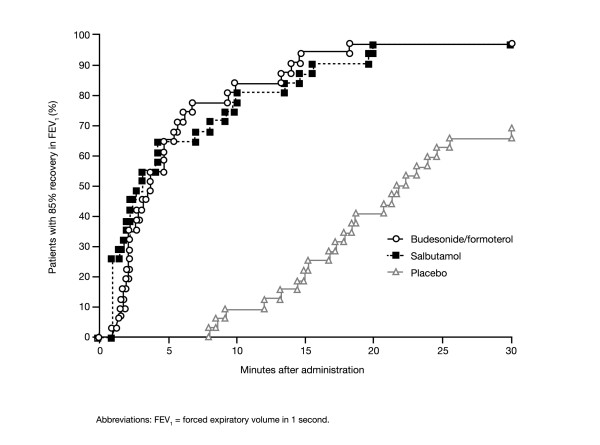
Kaplan-Meier plot for time to recovery to 85% of baseline FEV1 values obtained prior to induced bronchospasm in patients with asthma following one inhalation of one of the following as reliever medication: budesonide/formoterol 160/4.5 μg (via Turbuhaler^®^), salbutamol 100 μg (via pressurised metered-dose inhaler [pMDI]) or placebo. Reliever medication was given immediately after methacholine challenge.

The median time to recovery of FEV_1 _to 85% of baseline (which corresponds to approximately half the methacholine-induced fall) was similar for budesonide/formoterol and salbutamol (3.7 and 3.2 minutes, respectively; p = 0.1977; Figure 5), but significantly longer for placebo (22 minutes; p < 0.0001 for both budesonide/formoterol and salbutamol versus placebo) (Figure 5).

### Safety

Study procedures and treatments were well tolerated. Adverse events were few in number, of mild to moderate intensity and similar in reported pattern following all three test treatments. No serious adverse events were reported and no events led to study discontinuation. Adverse events often associated with β-adrenoceptor agonist therapy were few in number: palpitations were reported for one patient during salbutamol treatment; headache was reported for one patient receiving budesonide/formoterol and for one patient receiving placebo treatment.

## Discussion

The aim of this study was to compare the onset of relief of dyspnoea provided by a single dose of budesonide/formoterol with a standard two dose administration of salbutamol in an experimental human model of severe acute bronchoconstriction. Both budesonide/formoterol (160/4.5 μg one inhalation) and salbutamol (100 μg two inhalations) were superior to placebo for the primary efficacy variable – the onset of relief of dyspnoea, expressed as the change in Borg score 1 minute after study drug administration. A similarly rapid effect on FEV_1 _was observed at 1 minute after inhalation of the active treatments, confirming that patients perceive the rapid relief of bronchoconstriction with both active treatments (budesonide/formoterol and salbutamol) and that the perception of relief is mirrored by objective measurements of lung function.

Both budesonide/formoterol and standard salbutamol treatment resulted in rapid improvements in lung function within the first minutes after inhalation that returned to near baseline levels within 20 minutes. The median time to 50% recovery in dyspnoea and reversal of the fall in FEV_1 _were similar, approximately 2–3 minutes for both active treatments for dyspnoea and 3–4 minutes for FEV_1_, with no detectable or clinically relevant difference between the active treatments for either parameter. In contrast, both active treatments achieved reversal of the fall in FEV_1 _≥ 18 minutes ahead of placebo and dyspnoea relief ≥ 7 minutes ahead of placebo. Differences in Borg score and FEV_1 _after the first minute favoured the salbutamol regimen, but were not present thereafter. However, the effect of methacholine on Borg score and FEV_1 _was slightly less on the test days prior to budesonide/formoterol administration. Therefore, the window for recovery was slightly smaller for the combination therapy. Previous studies comparing formoterol and salbutamol showed similar effects for the two drugs [1-5]. Thus, the small differences seen at 1 minute in this study may in part be related to the study procedures and differences in baseline conditions.

Both budesonide/formoterol and salbutamol were well tolerated and the adverse events reported raised no safety concerns. The few adverse events that were reported were mild or moderate in intensity and occurred with a comparable frequency following placebo and active treatments.

The bronchoconstriction induced by methacholine is solely due to the contraction of airway smooth muscle cells and is, therefore, considered an appropriate model in which to investigate the smooth muscle relaxing effects of β_2_-agonists given as monotherapy or in ICS/LABA combinations. Both salbutamol and formoterol are known to reverse this contraction of airway smooth muscle that represents at least part of the component of airway obstruction occurring in an acute asthma exacerbation [1,2,26,27]. Two previous studies have shown that high-dose budesonide/formoterol was as effective and well tolerated in the treatment of acute asthma in an emergency setting as high-dose salbutamol [19] or high-dose formoterol [20]. The results of our study support the findings from these two previous high-dose studies and suggest that even a low formoterol dose administered as a single inhalation of budesonide/formoterol can be used to relieve severe asthma symptoms effectively.

Although available evidence points to a similar onset and magnitude of effect for salbutamol and formoterol (either as a single component or in the budesonide/formoterol combination) in reversing acute bronchoconstriction, formoterol and, particularly, formoterol/budesonide used as both maintenance and reliever therapy may have additional advantages over salbutamol. These advantages include improved asthma control through a longer duration of bronchodilation and bronchoprotection with formoterol [6,8] and a more timely adjustment in anti-inflammatory therapy with extra budesonide, given at the first sign of increasing symptoms [14,15,28,29].

## Conclusion

Budesonide/formoterol provides rapid relief of dyspnoea in asthma patients with experimentally induced bronchoconstriction. The relief of bronchoconstriction as perceived by asthma patients treated with budesonide/formoterol, confirmed by relief of objective lung function assessments, was similar to that observed with salbutamol. This suggests that the budesonide/formoterol combination is suitable for the immediate relief of asthma symptoms.

## Competing interests

The study described in this manuscript was supported by AstraZeneca, who also paid the article-processing charge. The Departments of Pulmonary Diseases at the Academic Medical Centre, Amsterdam (RE Jonkers) and Martini Hospital, Groningen (R Aalbers) received unrestricted research grants from AstraZeneca for the conductance of two clinical studies. R Aalbers has provided consultancy services to AstraZeneca, GlaxoSmithKline, Merck Sharp & Dohme and Novartis

## Authors' contributions

RE Jonkers, TA Bantje and R Aalbers were involved in the design of the study, data collection, analysis and interpretation and the drafting of the paper. All authors had complete access to the study report, made final decisions on all aspects of the article and hence are in agreement with, and approve, the final version of the submitted article.
